# Quality of insulin obtained from hospital and community pharmacies in Mwanza Tanzania: A cross-sectional study

**DOI:** 10.1371/journal.pone.0325366

**Published:** 2025-06-04

**Authors:** Emmanuel Kimaro, Eveline T. Konje, Benson R. Kidenya, Amani T. Mori, Eliangiringa Kaale

**Affiliations:** 1 Catholic University of Health and Allied Sciences (CUHAS), School of Pharmacy, Department of Pharmaceutics and Pharmacy Practice, Mwanza, Tanzania; 2 Catholic University of Health and Allied Sciences (CUHAS), School of Public Health, Department of Biostatistics, Epidemiology and Behavioral Sciences, Mwanza, Tanzania; 3 Catholic University of Health and Allied Sciences (CUHAS), Weill Bugando School of Medicine, Department of Biochemistry and Molecular Biology, Mwanza, Tanzania; 4 Department of Global Public Health and Primary Care, Section for Ethics and Health Economics, University of Bergen, Bergen, Norway; 5 Pharm R&D Laboratory, School of Pharmacy, Muhimbili University of Health and Allied Sciences (MUHAS), School of Pharmacy, Dar es Salaam, Tanzania; 6 Muhimbili University of Health and Allied Sciences (MUHAS), School of Pharmacy, Department of Medicinal chemistry, Dar es Salaam, Tanzania; Assistance Publique - Hôpitaux de Marseille, FRANCE

## Abstract

**Background:**

Insulin, a peptide hormone crucial for diabetes management, necessitates strict storage (2-8^o^C) to maintain concentration, as per regulatory guidelines (e.g., USP, WHO). Degradation can occur during manufacturing process, and transportation or storage, via heat-induced denaturation and aggregation, compromises its efficacy. In Sub-Saharan Africa, including Tanzania, weak regulatory oversight of imported medicines may compromise insulin quality. Varied findings from existing studies underline the need for local quality assessments.

**Methods:**

This cross-sectional study, conducted from April to July 2024 in Mwanza, Tanzania, evaluated the quality of insulin from eight pharmacies. A convenience sampling approach was employed, with insulin samples purchased as a simulated patient to reflect real-world acquisition. 15 samples, representing seven distinct insulin brands, were collected. High-Performance Liquid Chromatography (HPLC) was utilized to quantify insulin concentration. Additionally, samples were assessed for visual appearance, packaging, labeling, and storage conditions following established pharmacopoeial and quality control standards.

**Results:**

Out of 15 insulin samples, two samples (13.3%) failed to meet the official USP monograph during the assay test. The remaining samples 13 (86.7%) complied with USP standards.

**Conclusion:**

These findings underline significant challenges in ensuring the quality of insulin products. While most samples adhered to USP standards, the issues observed highlight the need for improved quality control measures and robust cold chain management to guarantee the efficacy and reliability of insulin for diabetic patients.

## Background

Insulin is a polypeptide hormone primarily produced by beta cells in the islets of Langerhans of the pancreas. It plays a crucial role in carbohydrate metabolism, and individuals who cannot produce insulin are unable to regulate their blood glucose levels effectively [1]. Insulin is a life-saving medication for individuals with type 1 diabetes mellitus and is also essential for some patients with type 2 diabetes mellitus who require daily injections to maintain glycemic control [2].

To preserve insulin’s concentration and efficacy, manufacturers and regulatory agencies such as the U.S. Food and Drug Administration (FDA), European Medicines Agency (EMA), and World Health Organization (WHO) recommend that all insulin formulations be stored within a temperature range of 2°C to 8°C (36°F to 46°F) [3]. This temperature range is specified in pharmacopeial monographs, such as the United States Pharmacopeia (USP) and European Pharmacopeia (Ph. Eur.), which outline stability requirements and quality control parameters for insulin storage and distribution [4]. However, in many Low- and Middle-Income Countries (LMICs), including Tanzania, maintaining these optimal storage conditions remains a challenge due to unreliable electricity supply, inadequate refrigeration facilities, and economic constraints [5]. Inadequate storage conditions have been widely recognized as contributors to potential insulin degradation. Nonetheless, an often overlooked but equally important issue is the source and manufacturing quality of insulin products, especially in LMICs where regulatory oversight of imported pharmaceuticals may be limited. Defective products may originate from manufacturers with poor quality control standards, making it difficult to attribute reduced concentration solely to storage conditions.

Insulin stability is affected by temperature fluctuations, direct exposure to light, and mechanical stress due to agitation, all of which can accelerate degradation processes. Degradation mechanisms include chemical degradation (e.g., hydrolysis and deamidation) and physical instability (e.g., fibrillation and precipitation) [6,7]. Regulatory guidelines like USP monograph, European pharmacopeia (Ph. Eur.), and ICH Q5C-stability of biotechnological/Biological products highlight that insulin formulations degrade rapidly at pH values below 5 and above 8, necessitating stringent control over formulation conditions to ensure product stability [8].

Different insulin formulations exhibit varying degrees of stability due to differences in peptide structure, excipients, and manufacturing processes [9]. Regulatory authorities require stability testing for each insulin formulation to establish shelf-life specifications under both recommended and stressed conditions. The International Conference on Harmonisation (ICH) guideline Q1A (R2) provides a framework for evaluating stability and defining appropriate storage conditions [10]. However, limited data exist on the quality of insulin formulations available in sub-Saharan Africa, necessitating region-specific studies to assess potential degradation risks.

In many parts of sub-Saharan Africa, including Tanzania, weak supply chain infrastructure coupled with limited regulatory capacity contributes to the importation of substandard medicines. Studies have shown that temperature excursions beyond the recommended range during transportation and storage can lead to chemical and physical degradation, reducing insulin’s content and clinical effectiveness [6]. Moreover, regulatory oversight of manufacturing facilities that supply imported medicines to these regions remains limited, with insufficient enforcement of pharmacovigilance measures to ensure insulin quality throughout the supply chain.

Several studies across Africa have highlighted significant concerns regarding insulin quality defects, primarily due to improper storage conditions and handling practices. For instance, research conducted in Ghana and Nigeria found that insulin vials stored in health facilities often exceeded recommended temperature limits, raising concerns about compromised potency [11,12]. In LMICs, evidence regarding insulin quality is scarce. One study conducted in Kenya found no significant change in insulin content after exposure to simulated laboratory conditions [6]. To address this gap and ensure insulin quality assurance in Tanzania, our study evaluated the quality of insulin obtained from community and hospital pharmacies in Mwanza, Tanzania.

## Methods

### Study design, duration and setting

This cross-sectional, laboratory based study was conducted from April to July 2024, in Mwanza city. Mwanza’s proximity to four international borders [Kenya, Uganda, Rwanda, and Burundi} underlines its significance for this study because pharmaceutical products including insulin can easily move across the porous borders. The presence of the Tanzania Medicines and Medical Devices Authority (TMDA) laboratory facility in Mwanza’s lake zone also made it suitable location for conducting this research. TMDA is classified as a Level 3 regulatory authority according to the World Health Organization (WHO) Global Benchmarking Tool (GBT) standards. A Level 3 classification indicates that the regulatory system has reached a level of maturity where it functions well and complies with most international standards for overseeing medical products. It also signifies that TMDA is capable of ensuring the safety, quality, and efficacy of medicines and medical devices.

### Sample size, sampling procedure, and selection criteria

A total of 15 human insulin samples, representing seven different brands, were collected from seven community pharmacies and one hospital pharmacy. A convenient sampling technique was employed to select pharmacies with high patient traffic and significant insulin sales. The insulin samples were obtained by purchasing them as regular customers without prior notification to the pharmacies. This approach ensured the assessment of insulin quality as it is supplied to patients. Similar sampling methods have been suggested in pharmaceutical surveillance studies [13,14]. We included samples that had complete labeling information and were within their shelf life. Insulin products in poor condition, such as those with leakage were excluded from the study.

### Pharmacy storage condition

The study utilized bluetooth temperature data logger device (LogTag Recorder Ltd, LogTag TRED30−7, New Zealand). Key specifications include: Measurement Range: −20°C to +40°C, with an accuracy of ±0.5°C. Battery Life. Designed for continuous operation for over 30 days, aligning with the study duration. Storage Capacity: Internal memory capable of storing approximately 45,000 data points, recorded at 15-minute intervals.

#### Deployment and removal protocol.

Placement: Pharmacists working in the pharmacies were requested to position the data loggers near their insulin storage locations, such as the refrigerator.

Monitoring Duration: Each device remained in place for 30 days to capture continuous temperature variations.

Retrieval: After the monitoring period, pharmacists returned the loggers to the research assistant. Data extraction was performed using Credo, which facilitated the transfer of recorded data to a computer equipped with the appropriate software for analysis.

#### Data mining and processing workflow.

Data Transmission: The Credo system was used to retrieve data from the loggers and transfer it to a dedicated computer for analysis.

Data Analysis: Temperature profiles were evaluated for deviations from recommended storage conditions: Refrigeration: 2°C to 8°C while for the room temperature: Below 30°C

### Material and reagents

Reagents that were used were all HPLC grade; Human insulin CRS batch 5 (67081 Strasbourg (France), Water (Sigma-Aldrich, Milli-Q water purification system, United States), orthophosphoric acid and sodium anhydrous sulphate (Sigma-Aldrich, analytical grade, United States), Acetonitrile (Sigma-Aldrich, analytical grade, United States). Apparatus that were used include; measuring cylinder (Pyrex Measuring Cylinder (Make: Pyrex, United States), volumetric flask (Class A Volumetric Flask (Make: Duran, Germany), beaker (Beaker: Pyrex Beaker (Make: Pyrex, United States), thermometer (H–B Instrument Thermometer (Make: H–B Instrument, United States), analytical scale (Mettler Toledo Analytical Balance (Make: Mettler Toledo, Switzerland).

### Equipment’s

HPLC machine (Model: Agilent 1260 Infinity II HPLC System: Agilent Technologies, United States) which was used for quantification of human insulin from samples collected. PH meter (Mettler Toledo Seven Compact S220 pH Meter Make: Mettler Toledo, Switzerland) for measuring PH, sonicator (Branson SFX150 Sonifier Make: Branson, United States) for sonication. Bluetooth Temperature Data Logger Device (LogTag Recorder Ltd, LogTag TRED30−7, New Zealand).

### System suitability test and identification test

System suitability solution of 1.5 mg/ml of human insulin in 0.01 N hydrochloric acid was prepared and allowed to stand at room temperature for Not less than three days ([NLT 3 days) to obtain a solution containing NLT 5% of A-21 desamido insulin human. The prepared solution was injected to HPLC and the mobile phase was prepared according to the USP monograph. The system suitability requirements are: Peak resolution NLT 2.0 between insulin human and A-21 desamido insulin human, system suitability solution. Tailing factor not more than (NMT 1.8). The retention time of the major peak of the sample solution corresponds to that of the standard solution, as obtained in the assay it will confirm the identity.

### Assay test

An assay test is an analytical method used to assess the purity, and content of a particular substance within a sample. It involves analyzing the sample’s properties, such as its chemical composition, and comparing them to a known standard to ensure quality and adherence to established specifications. In the current study we determined the insulin content. The analytical procedure for content determination was carried out according to the USP monograph [4].

### Ethics approval and consent to participate

This study received ethical approval from the CUHAS/BMC RESEARCH COMMITTEE (CREC), reference number (CREC/746/2024). No informed consent was needed as there were no human participants involved in the data collection process.

## Results

### Pharmacy storage condition

The study found that all wholesalers failed to follow cold chain transport guidelines, with no use of temperature loggers during receipt or distribution. Some also sourced insulin from street vendors, compromising quality. Temperature monitoring was conducted in eight pharmacies during period of data collection. Findings showed that during the study period, temperatures remained within the recommended range, with a mean of the Mean Kinetic Temperature of 6.052 (minimum 5.053, maximum 8.348). Mean Kinetic Temperature (MKT) was calculated to confirm that recorded temperatures were consistently within the acceptable limits throughout the data collection period.

### System suitability

The percentage Relative Standard Deviation (%RSD) values for peak area and retention time were within 2% as shown in Table 1 below, indicating the system’s suitability. The efficiency of the column separation, expressed by a resolution of NLT 2.0, and the peak symmetry, expressed by a tailing factor of 1.10 ± 1.1% (mean ± % RSD), also confirmed the system’s suitability.

**Table 1:** System suitability.

SN	Description	Mean peak area	Mean tailing factor (NMT 1.8)	Resolution (NLT 2.0)
1	Human insulin	13.76	1.10	13.01
2	% RSD	1.40	1.10	–

– Was not obtained.

Fifteen different insulin samples were used to assess the quality of human insulin as shown in Table 2. All brands were registered

**Table 2:** Commercial product information and insulin concentration in a different insulin brands.

SN	Brand Name	Generic Name	Batch No	Registration Status	Manufacturing country	Manufacturing date	Expiry date	(USP Specification NLT 27.5 mg) Amount, mg	(USP Specification 95%−105%) concentration	%RSD	Remarks
1	Insu 001	Human insulin	NT69S57	Registered	France	Sep/2022	Feb/2025	29.6 mg	103.4%	1.2%	Complies
2	Insu 002	Human insulin	NT6AG58	Registered	France	Mar/2023	Aug/2025	29.7 mg	104%	1.1%	Complies
3	Insu 002	Human insulin	NT6AG58	Registered	France	Mar/2023	Aug/2025	30.3 mg	105.6%	1.4%	Complies
4	Insu 002	Human insulin	NT6AG58	Registered	France	Mar/2023	Aug/2025	30 mg	105.1%	1.3%	Complies
5	Insu 003	Human insulin	MT67L55	Registered	France	Sep/2023	Dec/2024	30.1 mg	105.4%	1.3%	Complies
6	Insu 004	Human insulin	TB6080224	Registered	India	Feb/2024	Jan/2026	30 mg	105.1%	1.2%	Complies
7	Insu 005	Human insulin	TB409112	Registered	India	Nov/2022	Oct/2024	29.2 mg	102.1%	1.1%	Complies
8	Insu 006	Human insulin	TOB230723	Registered	India	Jul/2023	Jun/2025	29.9 mg	104.3%	1.0%	Complies
9	Insu 007	Human insulin	TB6040523	Registered	India	May/2023	April/2025	28.2 mg	98.5%	1.3%	Complies
10	Insu 008	Human insulin	BF23000689	Registered	India	Mar/2023	Feb/2026	30.3 mg	105.6%	1.2%	Complies
11	Insu 009	Human insulin	BF23000685	Registered	India	Mar/2023	Feb/2026	0 mg	0%		Does not complies
12	Insu 009	Human insulin	BF23000685	Registered	India	Mar/2023	Feb/2026	3.5 mg	12.2%	1.3%	Does not complies
13	Insu 009	Human insulin	BF23000685	Registered	India	Mar/2023	Feb/2026	4.4 mg	15.4%	1.1%	Does not complies
14	Insu 009	Human insulin	BF23000685	Registered	India	Mar/2023	Feb/2026	4.4 mg	15.4%	1.2%	Does not complies
15	Insu 010	Human insulin	BF21003364	Registered	India	Sep/2021	Aug/2024	0 mg	0%		Does not complies
16	Insu 011	Human insulin	BF22003678	Registered	India	Oct/2022	Sep/2025	30.2 mg	105.4%	1.1%	Complies
17	Insu 012	Human insulin	NT6AS79	Registered	France	May/2023	Oct/2025	29.6 mg	103.4%	1.4%	Complies
18	Insu 012	Human insulin	NT6AS79	Registered	France	May/2023	Oct/2025	29.6 mg	103.4%	1.2%	Complies
19	Insu 013	Human insulin	NT6BX88	Registered	France	Oct/2023	Mar/2026	29.6 mg	103.4%	0.8%	Complies
20	Insu 014	Human insulin	NT69V14	Registered	France	Mar/2023	Aug/2025	30 mg	105.1%	1.2%	Complies
21	Insu 012	Human insulin	NT6AS79	Registered	France	May/2023	Oct/2025	29.7 mg	104%	1.2%	Complies
22	Insu 015	Human insulin	NT69B80	Registered	France	Nov/2022	Apr/2025	30 mg	105.1%	1.1%	Complies

### Identification of insulin in products

**Fig 1:**
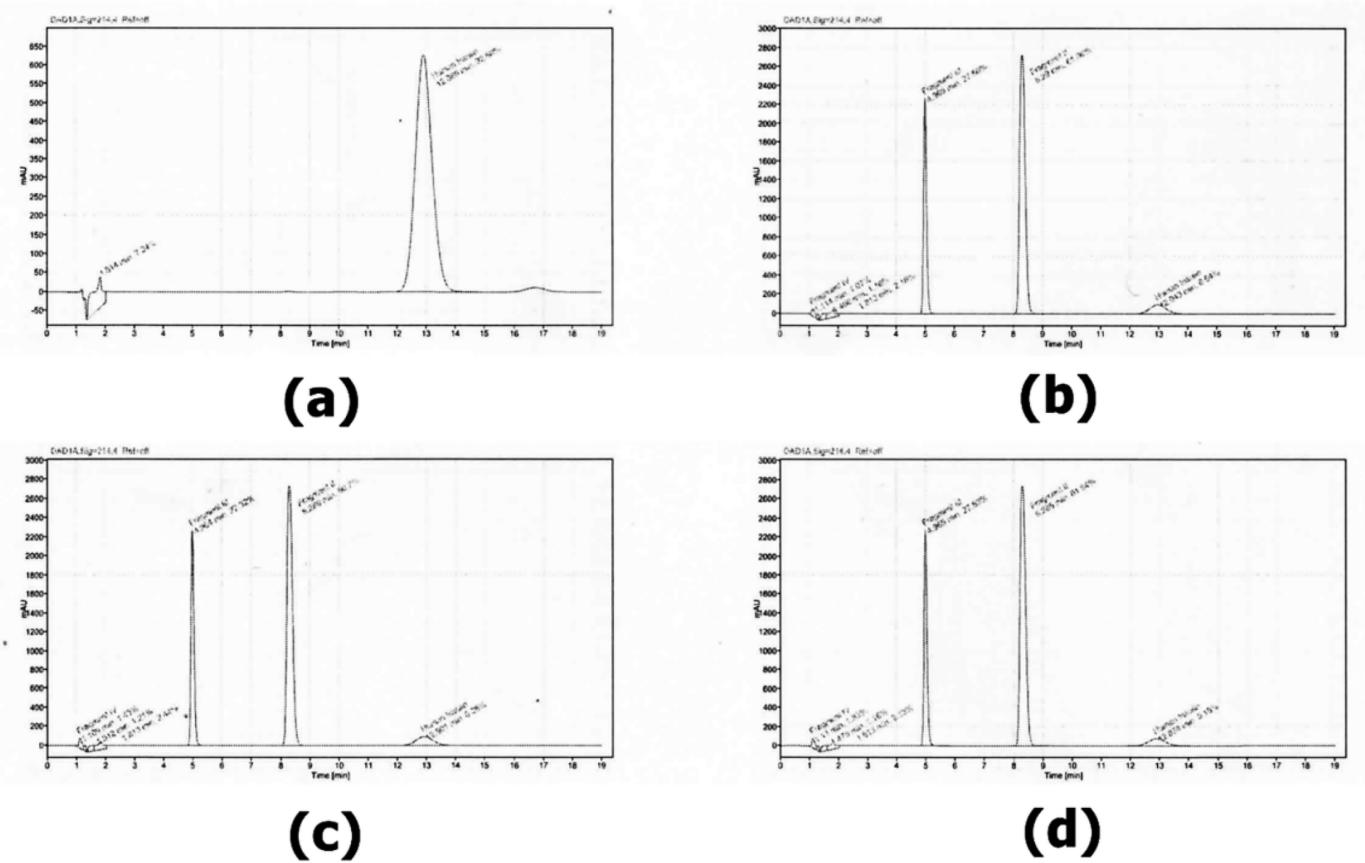
Chromatograms for the samples and reference standard solution of human insulin: “a” shows the reference standard solution of human insulin CRS, and “b,” “c,” and “d” show Insu 009 (batch no. BF23000685).

**Fig 2:**
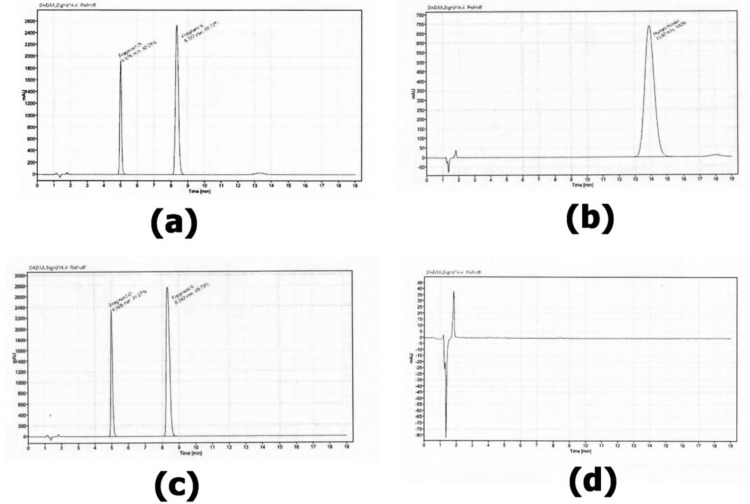
Chromatograms for the samples: “a” shows Insu 009 (batch no. BF23000685), “b” shows a standard solution of human insulin CRS, “c” shows 010 (batch no. BF21003364), and “d” is a blank with 0.01 N HCl.

The retention time of the major peak of the sample ranges from 12.8 minutes to 14.0 minutes, with a mean retention time of 13.1 minutes and a percentage relative standard deviation (%RSD) of 1.4%. This corresponds to the retention time of the reference standard solution, which is 13.7 minutes with an RSD of 1.4%. Fig 1 confirms the presence of human insulin in the injected samples, as evidenced by peaks corresponding to the insulin standard. In contrast, Fig 2 shows no detectable human insulin, suggesting either degradation or absence of the compound in those samples. Each set of figures includes a chromatographic peak for the reference standard (Figs 1a and 2b), which serves to confirm the identity of insulin in the samples. The corresponding sample peaks are used to both verify the presence of insulin and determine its concentration by comparison with the standard.

### Insulin concentration in products

Out of the 15 samples, 2 (13.3%) did not comply with USP specifications (not less than 27.5 mg) regarding the human insulin concentration in the vials, as shown in Table 2 above. The % RSD for reference standard was 1.1%

## Discussion

The temperature storage conditions for the insulin samples during the study period were satisfactory. Therefore, the storage temperatures in the pharmacy premises do not appear to explain the out-of-specification results observed in two samples (13.3%).

In the current study we found human insulin samples matched the reference standard solution in terms of retention time (13 minutes), with tailing factors not exceeding 1.8 and peak resolution not less than 2.0. These results indicate that the method used for detection and assessing insulin concentration in the vials was suitable for separating human insulin and A-21 desamido insulin. These findings are consistent with another study that determined the identification of human insulin in various pharmaceutical products, where the retention time was found to range between 14 minutes and 20 minutes [15,16].

Of the 2 (13.3%) non-compliant or substandard human insulin samples as shown in Table 2. This discrepancy could be attributed to poor manufacturing processes or poor handling during transportation. Manufacturing of large molecules like proteins (insulin) is very complex with many parameters that are difficult to control. Manufacturers of large molecules face significant challenges due to batch-to-batch variability, which can lead to final products that do not meet minimum quality standards [17,18]. The World Health Organization, U.S., and EU have developed guidelines for the distribution of cold chain products like insulin, placing responsibility on manufacturers, regulatory authorities, and wholesalers to ensure proper transport conditions [3].

Out of 15 human insulin samples tested, two were substandard (13.3%) failed to meet the official USP monograph standards in the assay test. This non-compliance likely stems from cold chain distribution challenges common in Sub-Saharan Africa, including Tanzania, as well as potential manufacturing shortcomings. These findings underline the urgent need to strengthen quality control and distribution systems for insulin products.

The remaining human insulin samples comply with official USP standards, meeting the acceptance criteria of not less than (NLT) 27.5 mg and concentration range of 95%−105% [4]. Out of 15 human insulin samples, 13 (13.3%) comply with the official USP standards as shown in the Table 2 above, consistent with findings from other studies [6,19,20]. The chromatograms in Figs 1 and 2 indicate that the human insulin samples (Insu 009 & 010) were substandard, likely due to poor formulation during manufacturing process or environmental factors such as temperature, light, and agitation during transportation.

Insulin monomers are highly susceptible to mechanical agitation during manufacturing, transportation, and handling. Agitation induces partial unfolding of the insulin structure, exposing hydrophobic regions of the peptide that are usually buried within the native conformation [21]. These exposed hydrophobic regions facilitate the self-association of insulin monomers, leading to aggregation. Over time, these aggregates can evolve into insoluble amyloid fibrils through a nucleation-dependent process [21]. This fibril formation not only reduces the active concentration of insulin in the vials but also compromises its therapeutic efficacy [22]. Furthermore, injecting insulin containing aggregates or fibrils can contribute to injection-site complications, including lipohypertrophy, lipoatrophy, and insulin-derived amyloidosis, while also impairing glycemic control [22]. The main limitation of this study is the lack of assessment for insulin fibrils in the non-compliant human insulin vials. We recommend further research on non-compliant insulin samples [23].

### Policy implications for insulin quality control in Tanzania

To address insulin quality issues in Tanzania, it is essential to strengthen quality control measures at all stages of the supply chain, including rigorous testing to ensure that insulin products meet active human insulin standards. Enhancing manufacturing processes and improving cold chain distribution infrastructure are crucial for maintaining insulin efficacy. Regular training programs for personnel working at Tanzania Medicine and Medical Device Authority (TMDA) so as to be identify sub-standard pharmaceutical products available in Tanzanian market, coupled with stricter regulatory oversight and enforcement, and are needed to uphold standards. Public awareness campaigns should educate stakeholders on proper insulin handling and storage, while collaboration with international partners can help to adopt best practices. Encouraging ongoing research and continuous improvement will ensure that policies and procedures remain effective, ultimately improving insulin quality and patient outcomes in Tanzania. Furthermore, WHO encourages integrating the supply chain for storing, handling, and distributing temperature-sensitive vaccines and non-vaccine [23].

## Conclusion

The study reveals significant insights into the quality of insulin products, highlighting that while most samples meet the official USP standards, a few do not, reflecting potential issues in manufacturing and distribution. Specifically, two samples lacked active human insulin, which may be due to inadequate manufacturing processes or improper storage during transportation.
